# Expression of the Arabidopsis WRINKLED 1 transcription factor leads to higher accumulation of palmitate in soybean seed

**DOI:** 10.1111/pbi.13061

**Published:** 2019-01-18

**Authors:** Pamela A. Vogel, Shen Bayon de Noyer, Hyunwoo Park, Hanh Nguyen, Lili Hou, Taity Changa, Hoang Le Khang, Ozan N. Ciftci, Tong Wang, Edgar B. Cahoon, Tom Elmo Clemente

**Affiliations:** ^1^ Center for Plant Science Innovation University of Nebraska‐Lincoln Lincoln NE USA; ^2^ Department of Agronomy & Horticulture University of Nebraska‐Lincoln Lincoln NE USA; ^3^ Center for Biotechnology University of Nebraska‐Lincoln Lincoln NE USA; ^4^ Department of Food Science & Technology University of Nebraska‐Lincoln Lincoln NE USA; ^5^ Department of Food Science and Human Nutrition Iowa State University Ames IA USA; ^6^ Department of Biochemistry University of Nebraska‐Lincoln Lincoln NE USA; ^7^ Present address: LG Chem Seoul Korea

**Keywords:** palmitic acid, high solids soybean oil, *Glycine max*

## Abstract

Soybean (*Glycine max* [L.] Merr.) is a commodity crop highly valued for its protein and oil content. The high percentage of polyunsaturated fatty acids in soybean oil results in low oxidative stability, which is a key parameter for usage in baking, high temperature frying applications, and affects shelf life of packaged products containing soybean oil. Introduction of a seed‐specific expression cassette carrying the *Arabidopsis* transcription factor WRINKLED1 (AtWRI1) into soybean, led to seed oil with levels of palmitate up to approximately 20%. Stacking of the AtWRI1 transgenic allele with a transgenic locus harbouring the mangosteen steroyl‐ACP thioesterase (GmFatA) resulted in oil with total saturates up to 30%. The creation of a triple stack in soybean, wherein the AtWRI1 and GmFatA alleles were combined with a FAD2‐1 silencing allele led to the synthesis of an oil with 28% saturates and approximately 60% oleate. Constructs were then assembled that carry a dual FAD2‐1 silencing element/GmFatA expression cassette, alone or combined with an AtWRI1 cassette. These plasmids are designated pPTN1289 and pPTN1301, respectively. Transgenic events carrying the T‐DNA of pPTN1289 displayed an oil with stearate levels between 18% and 25%, and oleate in the upper 60%, with reduced palmitate (<5%). While soybean events harboring transgenic alleles of pPTN1301 had similar levels of stearic and oleate levels as that of the pPTRN1289 events, but with levels of palmitate closer to wild type. The modified fatty acid composition results in an oil with higher oxidative stability, and functionality attributes for end use in baking applications.

## Introduction

Soybean (*Glycine max* [L.] Merr.) is the largest feedstock for protein and second largest source of vegetable oil in the world. In 2017, the United States soybean harvest was estimated at 89.5 million acres, reaching a record high of 4.38 billion bushels (USDA: www.nass.usda.gov). Commodity soybean seeds accumulate close to 40% protein and 20% oil, with the latter providing close to 90% of the US vegetable oil production. The higher demand for soybean oil for food, feed, and industrial applications over the past decade, has called upon for the development of soybeans with higher oil and quality content. Altering carbon networks and fatty acid synthesis is an ongoing effort emphasizing targeted genetic approaches to enhance total oil, nutritional properties and end‐use functionality, in soybean breeding programs.

Soybean oil is composed of approximately 14% saturated fatty acids, 20% monounsaturated fatty acids and over 65% of polyunsaturated fatty acids (PUFAs). The high content of PUFAs contributes to poor oil oxidative stability. This outcome impacts soybean oils functionality for applications in food industry and engine performance when used for biodiesel production (Clemente and Cahoon, 2009). Increasing oil oxidative stability has been achieved by partial hydrogenation, shifting the fatty acid profile towards higher saturated and monounsaturated fatty acids (FA) and reduced PUFAs. However, the former process results in accumulation of so‐called trans fatty acids, which have been associated with cardiovascular diseases (Korver and Katan, 2006). In addition, hydrogenated oil displays altered viscosity and lubricity limiting its potential for biodiesel applications (Moser *et al*., 2007). As a means to circumvent the need for partial hydrogenation processes, to improve oxidative stability of soybean oil, numerous genetic approaches have been implemented (Clemente and Cahoon, 2009).

The Arabidopsis *WRINKLED1* (At*WRI1*) transcription factor was originally identified based on a *wri1* mutant displaying low activity of glycolytic enzymes that resulted in 80% reduction in seed oil content (Focks and Benning, 1998). *WRI1* encodes for a APETALA2/ethylene responsive element binding protein, which is involved in global regulation of key enzymes of carbon metabolism and fatty acid biosynthesis (Cernac and Benning, 2004; Ruuska *et al*., 2002). Further studies showed that At*WRI1* also plays a role in processes such as lipid assembly, storage, flowering time, seed development, embryo maturation, plant hormone regulation, and photosynthesis (Cernac *et al*., 2006; Kong *et al*., 2017; Li *et al*., 2015; Maeo *et al*., 2009; Wu *et al*., 2014). A number of other communications have also revealed orthologs of At*WRI1* being involved in plant oil biosynthesis (Adhikari *et al*., 2016; An *et al*., 2017; Bhattacharya *et al*., 2016; Chen *et al*., 2017; Ivarson *et al*., 2016; Kang *et al*., 2017; Kim *et al*., 2016; Liu *et al*., 2010; Ma *et al*., 2013; Pouvreau *et al*., 2011; Shen *et al*., 2010; Vanhercke *et al*., 2013; Wu *et al*., 2014; Yeap *et al*., 2017). For example, expression of the *Brassica napus WRI1*(*BnWRI1*) in *Arabidopsis* promoted between 10% and 40% increased seed oil content and led to larger seed and size mass (Liu *et al*., 2010). In rapeseed ectopic expression of *BnWRI1* enhances chlorophyll content in developing embryos, with increases observed in both oil and mass of mature seeds through the coordination of fatty acid biosynthesis and photosynthesis (Wu *et al*., 2014). Overexpression of the maize *ZmWRI1*, which shares 43% identity with the *AtWRI1*, translated to boost in kernel oil content by 30% across 15 events, when expressed under the embryo preferred OLE promoter (Shen *et al*., 2010). In addition, constitutive expression of *ZmWRI1a*, under control of the cassava vein mosaic virus promoter, also increased fatty acid content in maize kernels (Pouvreau *et al*., 2011). The oil palm, *Elaeis guineensis, EgWRI1* is capable of complementing the *wri1* mutant phenotypes in *Arabidopsis,* including seed oil, and restoration of off‐types in germination and seedling establishment (Ma *et al*., 2013). Transient co‐expression of *AtWRI1* and *AtDGAT* in tobacco leaves shifted polyunsaturated to monounsaturated fatty acids and increased TAG biosynthesis in leaves (Vanhercke *et al*., 2013). Under control of the seed specific napin promoter, the soybean *GmWRI1* increased oleic and linoleic leading to increased oil content in soybean (Chen *et al*., 2017).

Clearly *WRI1* acts as a regulator of oil biosynthesis, which when expression is perturbed, translates to changes in oil accumulation, with modest alterations in fatty acid composition. Approaches to impart significant changes in fatty acid profile of oil in seeds have included introduction of genes such as steroyl‐ACP thioesterase A (*FatA*), and fatty acid desaturase 2 (*Fad2*) can provide additional impacts on oxidative stability and functionality for margarine type applications, deep frying, or as liquid transportation fuel such as biodiesel. While the heterologous expression of a mangosteen (*Garcinia mangostana*) *GmFatA1*, led to substantial increases in stearic acid in rapeseed and soybean (Facciotti *et al*., 1999; Hawkins and Kridl, 1998; Park *et al*., 2014). Similarly, expression of the sunflower *FatA* in *Arabidopsis* led to an increase in both stearic and oleic acid content with a concomitant reduction in total oil (Moreno‐Pérez *et al*., 2013).

Decreasing the amount of polyunsaturated fatty acids, with a commitment boost in oleic acid, improves plant oil's oxidative stability and brings its acyl composition closer to that of olive oil. This increase in monounsaturated FA has been achieved through mutagenesis or down regulation of *FAD2* and *FAD3* desaturase genes in plants. Pham *et al*. (2010) showed that in soybean the combination of mutant alleles of both *FAD2‐1A* and *FAD2‐1B* was necessary to generate high (>80%) oleate lines through conventional breading. In addition, increased oleate has been achieved by the down‐regulation of *FAD2* through hairpin silencing in Arabidopsis (Stoutjesdijk *et al*., 2002), cotton (Liu *et al*., 2002), and through RNA silencing approaches in soybean (Buhr *et al*., 2002; Kinney and Knowlton, 1997; Park *et al*., 2014). The requirement for both seed specific homologues of *FAD2* in soybean to be down‐regulated to achieve high oleate levels was also verified through TALENs mediated editing (Haun *et al*., 2014).

Herein, is communicated the outcomes of the introduction of a seed‐specific *AtWRI1* expression cassette, under the control of the β‐conglycinin promoter in soybean, wherein increases palmitate, with negligible changes in total oil content was observed. Furthermore, as a means to increase total saturates and reduce PUFAs, crosses were made to produce a gene stack of *AtWRI1* with *GmFATA* (Park *et al*., 2014), and subsequently a triple stack assembled in soybean by combining, via crossing, the transgenic alleles of *AtWRI1, GmFATA,* with a FAD2‐1 silencing element (Buhr *et al*., 2002). The phenotypic outcomes, in seed oil fatty acid changes, triggered by these transgene stacks were recapitulated by assembling an expression cassette, carrying a strategic design, under the control of a single promoter, to simultaneously down‐regulate *FAD2‐1* gene and express *GmFATA* transgene. This dual functioning genetic element was introduced into soybean alone, or in combination with the *AtWRI1* cassette.

## Results

### Expression of the Arabidopsis *AtWRI1* in soybean

**Figure 1:**
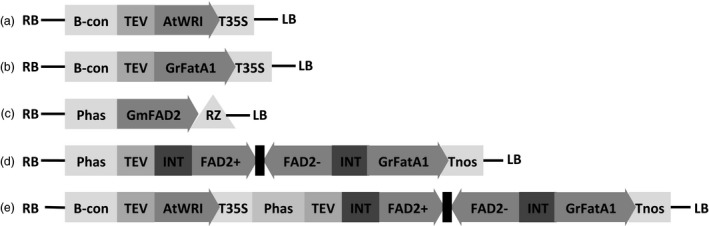
Diagram of the expression cassettes utilized in this study. (a) T‐DNA element of plasmid pPTN1174 harboring a coding region of the *AtWRI1* transcription factor from Arabidopsis under control of the beta‐conglycinin promoter. (b) T‐DNA element of plasmid pPTN811 harboring the steroyl‐ACP thioesterase A FatA1 coding region from *Garcinia mangostana* under the B‐conglyncine promoter. (c) T‐DNA elements of plasmid pPTN326 harboring *GmFad2‐1* coding region terminated by a self‐cleaving ribozyme designed for down‐regulation, under the common bean phaseolin promoter. (d) T‐DNA of plasmid pPTN1289 harboring a hairpin for *GmFad2‐1* embedded in an intron followed by the mangosteen *GmFatA1*, all under the control of the phaseolin promoter. (e) T‐DNA element of plasmid pPTN1301 which combines the *AtWRI1* expression cassette and the dual functional cassette from pPTN1289. RB: right border. LB: left border. B‐con: beta‐conglycinin promoter. Phas: phaseolin promoter. TEV: translational enhancer from tobacco etch virus. T35S: cauliflower mosaic virus 35S polyadenylation signal. Tnos: *Agrobacterium* nopaline synthase terminator. RZ: ribozyme. *AtWRI*: Wrinkled1 transcription factor from *A. thaliana. GmFatA1*: steroyl thioesterase from *Garcinia mangostana. GmFAD2*: fatty acid desaturase 2 from *Glycine max*. INT: intron.

As a means to increase total oil content in soybean seed, an expression cassette was assembled carrying a soybean codon optimized version of the *AtWRI1* (Figure 1a). The open reading frame (ORF) was fused to the tobacco etch virus translational enhancer element (TEV) (Carrington and Freed, 1990), and cloned downstream of the soybean seed‐specific β‐conglycinin promoter (Allen *et al*., 1989). The cassette was terminated with the 35S cauliflower mosaic virus polyadenylation signal. The final binary vector is designated pPTN1174, which carries a bar gene cassette (Thompson *et al*., 1987) for selection during the transformation process. The T‐DNA element of pPTN1174 was introduced into soybean via *Agrobacterium*‐mediated transformation (Xing *et al*., 2000; Zhang *et al*., 1999). Three independent events, referred to as 915‐25, 917‐17, and 917‐26 were selected for characterization. Complexity of the transgenic allele in the selected events was ascertained via Southern blot analysis (Figure S1).

The outcome of expressing the *AtWRI1* in soybean was accessed by monitoring the fatty acid profile and total oil content in soybean seeds through gas chromatography (GC‐FID). The GC‐FID data reveled no change in total oil in T_2_ seeds from plants grown under greenhouse conditions, however, a significant increase, between 36% and 69% above controls, in palmitate was observed (Table S1). This elevation in palmitate was accompanied by the concomitant reduction of oleate, which went from 17.5% in the WT down to 9% in the transgenic events (Table S1). As a means to determine if this elevated palmitate outcome translates to a field environment *AtWRI1* soybean events were grown under field conditions, near Mead, NE during the 2015, 2016, and 2017 seasons. Harvests from two homozygous lineages of events 915‐25 and 917‐17, along with WT were evaluated via GC‐FID. The results across the three consecutive years mirrored the greenhouse outcome, significant increase in palmitate, with no significant alteration in total oil content (Tables 1 and 2). Mirroring the greenhouse results, this elevation in palmitate came with a concomitant reduction in stearic, oleic, and linoleic acid (Table 1).

**Table 1:** Fatty acid profile of soybean seeds from field plants carrying AtWRI1

Event	Total Oil	16:0	18:0	18:1	18:2	18:3
WT	23.9 ± 0.6^a^	10.9 ± 0.1^b^	3.6 ± 0^a^	24 ± 0.6^a^	53.5 ± 0.5^b^	8.1 ± 0.1^a^
915‐25	22.8 ± 0.8^a^	18.3 ± 0.1^a^	2.5 ± 0^b^	14.8 ± 0.3^b^	57.8 ± 0.3^a^	6.7 ± 0.1^a^
917‐17	22.5 ± 0.8^a^	19.2 ± 0.4^a^	2.5 ± 0.1^b^	14.8 ± 0.3^b^	58.4 ± 1.2^a^	5.1 ± 1.7^a^
WT	23.6 ± 0.8^a^	10.4 ± 0.1^b^	3.1 ± 0^a^	24.5 ± 0.4^a^	53.5 ± 0.3^b^	8.5 ± 0.1^a^
915‐25	25.2 ± 1.1^a^	19 ± 0.3^a^	1.9 ± 0^b^	15.1 ± 0.4^b^	56.9 ± 0.4^a^	7.2 ± 0.1^b^
917‐17	26.5 ± 1.3^a^	18.9 ± 0^a^	2 ± 0^b^	16.1 ± 0.4^b^	56 ± 0.3^a^	6.9 ± 0.1^b^

Percentage of palmitic (16:0), stearic (18:0), oleic (18:1), linoleic (18:2), and linolenic acid (18:3), in soybean events harboring WRI1 (pPTN1174). Top: 2016. Bottom: 2017. Plants were grown under field conditions. Seven seeds per plant from two plants per plot and two plots were evaluated. Data expressed as mean ± standard error (*n* = 2 plots). Different letters indicate statistical differences (*P* < 0.05) within events.

**Table 2:** Fatty acid profile of soybean events and stacks evaluated under field conditions

Gene	Event	16:0	18:0	18:1	18:2	18:3
WT	WT	10.6 ± 0.1	3.8 ± 0.1	21.7 ± 0.2	52.2 ± 0.2	8.6 ± 0.0
AtWRI	915‐25	18.1 ± 0.1*	2.7 ± 0.0*	13.8 ± 0.0*	55.8 ± 0.2*	6.9 ± 0.0*
GmFATA1	683‐2	7.6 ± 0.0*	17.7 ± 0.1*	16.1 ± 0.2*	46.3 ± 0.2*	8.6 ± 0.1*
RNAi FAD2‐1	374‐1	7.1 ± 0.0*	3.6 ± 0.0	73.9 ± 0.2*	5.4 ± 0.0*	6.3 ± 0.0*
GmFATA1 × WRI	683‐2 × 915‐25	12.2 ± 0.0*	15.5 ± 0.1*	14.5 ± 0.1*	47.2 ± 0.2*	6.7 ± 0.1*
RNAi FAD2‐1 × GmFATA1	374‐1 × 687‐2	4.9 ± 0.0*	12.1 ± 0.1*	67.7 ± 0.3*	5.1 ± 0.0*	6.1 ± 0.1*
RNAi FAD2‐1 × GmFATA1 × WRI	374‐1 × 683‐2 × 915‐25	9.6 ± 0.4*	18.7 ± 0.9*	57.4 ± 0.8*	4.6 ± 0.3*	4.8 ± 0.3*

Percentage of palmitic (16:0), stearic (18:0), oleic (18:1), linoleic (18:2), and linolenic acid (18:3) in soybean seeds harboring AtWRI1 (pPTN1174), mangosteen GmFatA1 (pPTN811), RNAi GmFad2 (pPTN326), double and triple stacks derived by crossing. 40 seeds per event/stack per plot were bulked and analysed using methanolization for FAME synthesis. Data indicate mean ± stdev (*n* = 3 plots).

* Statistical differences across events. *P* < 0.001.

### Stacking of *AtWRI1* with the mangosteen *FatA1* and *GmFad2* silencing element

As a means boost oxidative stability, while lowering melting temperature of soybean oil, a set of transgene stacks were generated via crossing. To this end, the *AtWRI1* events 915‐25, and 917‐17 were crossed events 683‐2 and 687‐2, which carry a stearoyl ACP thioesterase *FatA1* from *Garcinia mangostana* (Park *et al*., 2014). In addition, an event designated 374‐1, which carries a silencing element targeting *Fad2* gene, was also used in the crossing scheme (Buhr *et al*., 2002) (Figure 1a–c). The rationale for stacking these transgenic alleles being the outcome is expected to result in an oil, high in saturated fatty acids, coupled with elevated oleic acid, and reduced PUFAs. An oil with such a fatty acid profile would possess functionality in baking applications and have improved performance as a biodiesel in warm climates. Here, multiple crosses were generated and F_2_ seeds were phenotyped for fatty acid composition (Table S2). Stacking of *AtWRI1* with *GmFatA1* alleles leads to higher accumulation of palmitate and stearate, which ranged between 16%–18%, and 8%–12%, respectively. Stacking the *GmFad2* silencing element with *GmFatA1* allele led to increased stearate and oleate by 65%, and sixfold, respectively. However, this combination results in a significant reduction in palmitate levels. The triple stack wherein all three transgenic alleles were combined, displayed increased stearic and oleic acid while restoring palmitate closer to wild‐type levels, relative to the palmitate levels observed in the GmFaad2 silencing/GmFatA1 stack (Table S2). Populations derived from select crosses were carried on to homozygosity under greenhouse conditions.

Homozygous lineages (F_4_ generation) were subsequently evaluated under field conditions in 2015 (Table 2). Fatty acid composition of both the two and triple stacks, along with corresponding parental events are shown in Table 2. The phenotypic outcomes, relative to those observed under greenhouse conditions (Table S2), mimic the field environment, with respect to directional changes in saturated and unsaturated fatty acid percentages of the oil. For instance, bulk seeds from the double stack 374‐1 × 687‐2, which combines the transgenic alleles of *GmFad2* silencing element with the mangosteen *GmFatA1*, display levels of stearate and oleate up to 12.1% and 67.7%, respectively, while palmitate is reduced to 4.9%. However, introgressing of the *AtWRI1* transgenic allele to create the triple stack, ‘374‐1 × 683‐2 × 915‐21’, boosts palmitate levels, along with stearate up to 18.7%, and 57.4% in oleate (Table 2).

### Increasing total saturates and oleic acid in soybean oil with a single expression cassette

As a means to increase total saturates and oleic acid while reducing the number of genetic elements introduced into soybean genome, an alternative genetic design was developed. To this end, a single cassette that targets the simultaneous down‐regulation of *Fad2*, and expression of the mangosteen *GmFatA1* transgene, alone or stacked with the *AtWRI1* cassette from pPTN1174 were assembled (Figure 1d,e). The former, designated pPTN1289, harbours a hairpin element imbedded in an intron targeting *GmFad2*, which resides 5′ to the TEV‐*GmFatA1* ORF fusion. This dual silencing/expression cassette is terminated by the nopaline synthase polyadenylation signal (Figure 1d). The latter genetic design, called pPTN1301, harbors the dual action cassette that resides in pPTN1289 stacked with *AtWRI1* from pPTN1174. The binary vectors were delivered into soybean via *Agrobacterium*‐mediated gene transformation (Xing *et al*., 2000; Zhang *et al*., 1999). Southern blot analysis on a subset of the derived transgenic soybean events carrying the respective T‐DNAs of pPTN1289 and pPTN1301 are shown in Figure S2.

Monitoring of fatty acid composition on seeds derived from soybean events carrying the T‐DNA element of pPTN1289 grown under greenhouse conditions revealed stearic and oleic acid up to 25, and 69%, respectively, in T_2_ populations (Table S4), mirroring the outcome of the crossing stack lineages carrying the *GmFatA1* and silenced *GmFad2* transgenic alleles. The increases in stearic and oleic acid came with the concomitant reduction in palmitic acid, which went from 12.2 in the wild‐type down to 3.8% in the transgenic events. In seed lineages derived from events carrying pPTN1301 T‐DNA, palmitic acid was restored to wild‐type levels due to the presence of the *AtWRI1*, in a similar fashion observed in the triple stack generated by crossing, coupled with stearic acid at approximately 22% and oleate reaching 65% (Table S4).

Three independent events harboring pPTN1289, and pPTN1301 were selected to determine if phenotypes observed under greenhouse conditions translated to a field environment. In the field trial, three plots per event, each consisting of 4 10‐ft rows, were planted 2016, and two plots per event were planted in 2017. Fatty acid profiles of seeds harvested from these trials are shown in Table 3. Results from two pPTN1301 events are not shown due apparent silencing. However, the phenotypes of the three pPTN1289 events and remaining pPTN1301 event were maintained under field conditions during 2016, and 2017, indicating that total saturates and oleic acid can be increased through a single expression cassette, and that *AtWRI1* increases palmitate in soybean.

**Table 3:** Fatty acid profile of pPTN1289 and pPTN1301 events grown in the field 2016 (Top), and 2017 (Bottom)

Plasmid	Event	Total oil	16:0	18:0	18:1	18:2	18:3
WT	Thorne	15.3 ± 1.9^a^	11.3 ± 0.2^b^	3.5 ± 0.04^de^	24 ± 0.4^c^	53.5 ± 0.4^a^	7.6 ± 0.1^b^
pPTN1289	1008‐5	16.2 ± 0.8^a^	4.8 ± 0.1^c^	25 ± 0.1^a^	66.1 ± 0.2^a^	1 ± 0.04^c^	3.1 ± 0.1^e^
pPTN1289	1022‐4	15.3 ± 0.8^a^	4.7 ± 0^c^	18 ± 0.4^c^	69.3 ± 0.5^a^	3.1 ± 0.2^c^	4.8 ± 0.1^c^
pPTN1289	1022‐15	13.9 ± 1.2^a^	4.2 ± 0^c^	20.6 ± 0.3^b^	69.2 ± 0.3^a^	2.1 ± 0.3^c^	3.9 ± 0.1^d^
pPTN1301	1026‐1	12.8 ± 0.5^a^	9.9 ± 0.2^b^	18 ± 0.3^c^	63.1 ± 0.3^a^	3.6 ± 0.1^c^	5.3 ± 0.1^c^
WT	Thorne	26.1 ± 1.6^a^	11.2 ± 0.1^a^	3.8 ± 0^b^	21.7 ± 0.8^d^	54.5 ± 0.7^a^	8.8 ± 0.2^a^
pPTN1289	1008‐5	15.1 ± 1.6 ^cd^	4.2 ± 0.1 ^cd^	16.4 ± 1.8^a^	71.2 ± 0.2^a^	2.9 ± 0.8^b^	5.3 ± 0.8^b^
pPTN1289	1022‐4	17.5 ± 1.1 ^cd^	4.7 ± 0.1^c^	15.1 ± 0.5^a^	70.9 ± 0.6^a^	3.4 ± 0.1^b^	5.9 ± 0.2^b^
pPTN1289	1022‐15	18.9 ± 1.1^bcd^	4 ± 0^d^	15.8 ± 0.2^a^	71 ± 0.3^a^	3.1 ± 0.1^b^	6.1 ± 0.3^b^
pPTN1301	1026‐1	14.8 ± 1^d^	9.9 ± 0.4^b^	12.5 ± 1^a^	61.4 ± 1.1^b^	9.5 ± 1.9^b^	6.6 ± 0.2^b^

Percentage of total oil, palmitic (16:0), stearic (18:0), oleic (18:1), linoleic (18:2), and linolenic acid (18:3), in soybean events harboring pPTN1289 (RNAi Fad2, GmFatA1), and pPTN1301 (RNAi Fad2, GmFatA1, AtWRI1) Plants were grown under field conditions, Top: 2016. Bottom: 2017. Seven seeds per plant (positive for PCR) were pooled for fatty acid analysis. Two plants per plot and three plots per event were used for data analysis. Data indicate mean ± sterr (*n* = 3). Different letters indicate statistical differences across events. *P* < 0.05.

### Elevated palmitate in seed oil triggered by *AtWRI1* expression is due to up‐regulation of a soybean *FatB* allele

**Figure 2:**
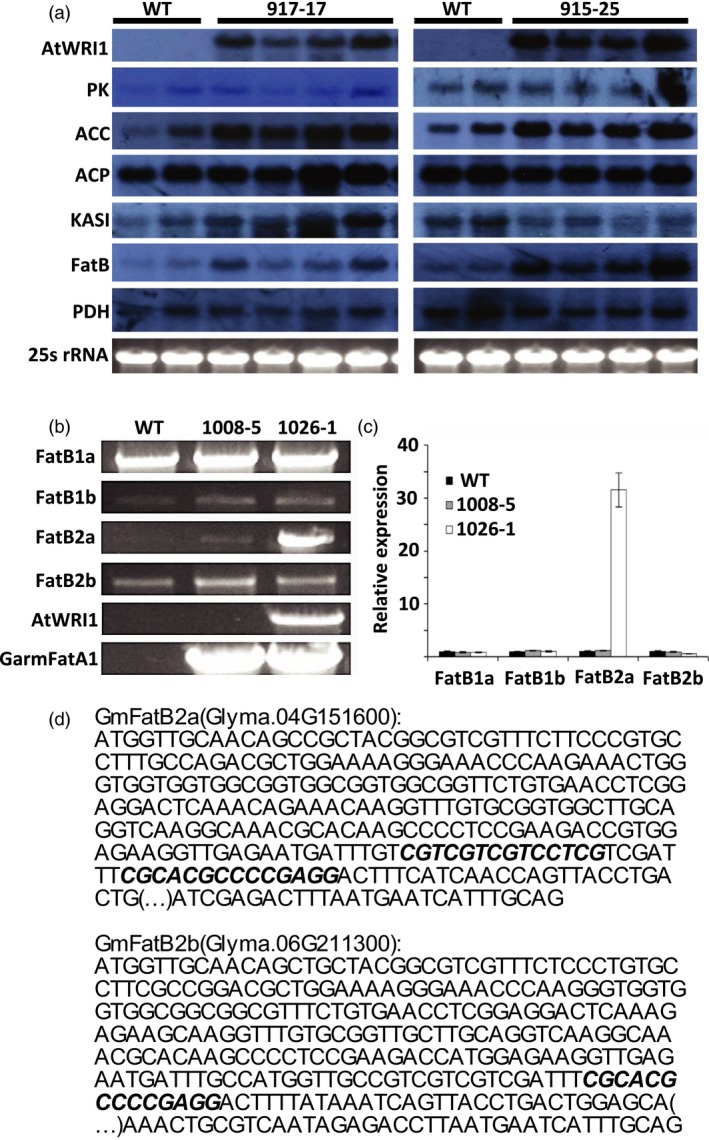
Expression analysis of *AtWRI1* target genes in soybean immature embryos. (a) northern blot analysis on WT, and transgenic events 917‐17, and 915‐25 (pPTN1174). Probes were used against gene sequences coding for *A. thaliana* WRINKLED1 (*AtWRI1*), and *G. max* genes: Pyruvate Kinase (PK), Acetyl‐CoA Carboxylase (ACC), Acyl Carrier Protein (ACP), β‐ketoacyl ACP synthase (KASI), Fatty Acid Thioesterase B (FatB), and Pyruvate Dehydrogenease (PDH). Ribosomal RNA (25S rRNA) is used to show equal RNA concentrations across WT and transgenic events. (b) RT‐PCR analysis of four FatB genes from soybean: FatB1a (Glyma.05g012300), FatB1b (Glyma.17g120400), FatB2a (Glyma.04g151600), FatB2b (Glyma.06g211300); AtWRI1, and mangosteen GarmFatA1 on WT, 1008‐5 (pPTN1289), and 1026‐1 (pPTN1301). (c) Fold change relative expression of four FatB genes from soybean: FatB1a (Glyma.05g012300), FatB1b (Glyma.17g120400), FatB2a (Glyma.04g151600), FatB2b (Glyma.06g211300), relative to actin on WT, 1008‐5 (pPTN1289), and 1026‐1 (pPTN1301) determined by qRT‐PCR. (d) Location in GmFatB2a and GmFatB2b Exon 1 of the AW‐box motif [CnGnT](n)_7_[CG] of the reverse complement of the motif [CG](n)_7_[CnAnG], where n can be any nucleotide, as described by Maeo *et al*. (2009).

**Figure 3:**
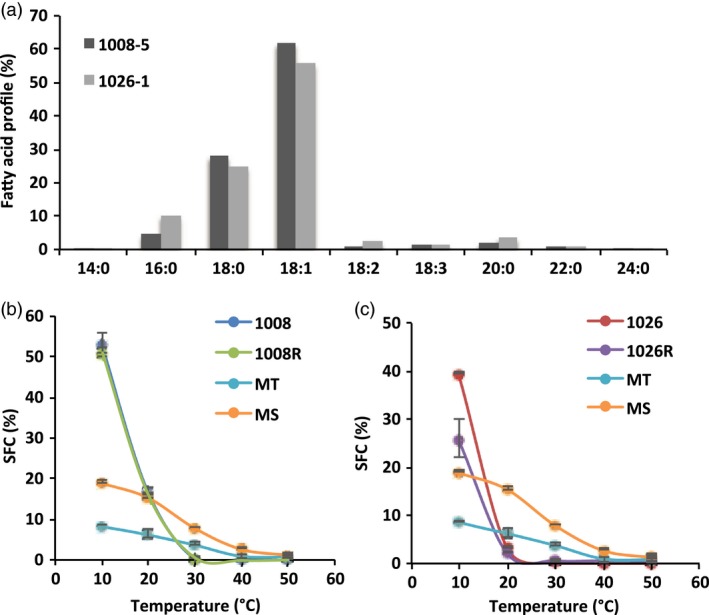
Physical properties of oils derived from pPTN1289, and pPTN1301. (a) Fatty acid profile derived from extruded oils from events 1008‐5, and 1026‐1 harboring pPTN1289, and pPTN1301, respectively. (b) Solid fat content percentage (SFC) of oil derived from the event 1008‐5 carrying pPTN1289 before, and after randomization (1008, 1008R, respectively), and tub (MT) and stick (MS) margarine. (c) Solid fat content percentage of oil derived from the event 1026‐1 carrying pPTN1301 before, and after randomization (1026, 1026R, respectively), and tub (MT) and stick (MS) margarine.

The WRI1 expression modulates the activities of target genes involved in glycolysis and fatty acid synthesis (Adhikari *et al*., 2016; Li *et al*., 2015; Maeo *et al*., 2009; Ruuska *et al*., 2002; Sanjaya *et al*., 2011; To *et al*., 2012), the expression of multiple genes in soybean seeds from two events (917‐17, and 915‐25) carrying pPTN1174 were determined via northern blots. To that end, endogenous transcript levels of the soybean homologs for pyruvate kinase (PI‐PKb1), acetyl‐CoA carboxylase subunit (BCCP2), acyl‐carrier protein (ACP1), β‐ketoacyl‐acp synthase (KASI), acyl‐ACP thioesterase B (FatB), and pyruvate dehydrogenase (PDH1α), as well as transgenic *AtWRI1* transcript were monitored (Figure 2a). Among the gene calls selected for transcript monitoring increased expression of *ACCase* and *FatB* is observed. These results indicate that the up‐regulation of *GmFatB* triggered by the transcription factor *AtWRI1* is the underlying mechanism boosting palmitate level of seed oil. To identify which of the four FatB alleles present in soybean genome are up‐regulated by *AtWRI1*, RT‐PCR, and qRT‐PCR analyses were conducted. Gene calls for *FatB1a*,* FatB1b*,* FatB2a*, and *FatB2b* (Cardinal *et al*., 2014), were amplified from cDNA isolated from event 1026‐1 seeds, which carries pPTN1301 along with two controls: WT (Thorne), and the event 1008‐5 which harbors pPTN1289 (Figure 3b,c). While levels of endogenous *FatB2a* were similar between WT and 1008‐5, the event 1026‐1 harboring *AtWRI1* displayed an increase in *FatB2a* by 30‐fold compared to the WT. No differences were observed in changes in expression of any of the other endogenous *FatB* gene calls. These results indicate that *AtWRI1* up‐regulates specifically *GmFatB2a* in transgenic events, leading to increases in palmitate in soybean seeds (Figure 3b,c).

### Physical and biochemical properties of oils derived from triple crosses

Oil extruded from selected events carrying the respective transgenic alleles *AtWRI1*,* GmFatA1*, silencing element for *GmFad2*, and triple stack were evaluated for impact on oxidative stability, and melting point. Fatty acid profiles of the evaluated extruded oils derived from the respective events, stacks and control are shown in Table 4. In most cases presence of the *AtWRI1* transgenic allele in the triple stack, be it on the same T‐DNA (i.e. pPTN1301; Tables 3, Table S4) or via crossing (Tables 2, Tables S2 and S3), palmitate levels tend to be 8.5% or greater. However, the triple stack plots harvested from the field in 2015 (Table 4) possessed palmitate levels of approximately 6.7%. While this lower palmitate level observed may be attributed to some silencing, and/or segregation in a lineage thought to be homozygous for AtWRI1 transgenic allele in the triple cross, the soybean oil from the triple stack displayed superior higher oxidative stability index (OSTI) relative to the other samples (Table 4). The high oleic acid sample derived from the silenced Fad2, event 374‐1, also showed improvement to this parameter, compared to control and the oil derived from events carrying the *AtWri1* and *GmFatA* alleles alone (Table 4). These data highlight the importance of reducing the level of polyunsaturated fatty acids in vegetable oil is key to boosting oxidative stability, while elevation in saturated fatty acids, coupled with high oleic acid leads to further increases in the OSTI.

**Table 4:** Fatty acid profile and physical properties of extruded oils from plants grown under field conditions in 2015

Gene	Sat	16:0	18:0	18:1	18:2	18:3	OSTI (h)	Melting Point (°C)
WT	14.09 ± 0.01	10.13 ± 0.01	3.97 ± 0.01	24.19 ± 0.02	52.04 ± 0.09	7.97 ± 0.01	6.6 ± 0.1	−30.3 ± 1.0
AtWRI	20.93 ± 0.03*	18.21 ± 0.01*	2.72 ± 0.03*	15.18 ± 0.03*	55.83 ± 0.18*	6.4 ± 0.03*	7.7 ± 0.9	−30.5 ± 0.6
GmFATA1	26.16 ± 0.05*	7.23 ± 0.01*	18.93 ± 0.04*	17.35 ± 0.01*	45.8 ± 0.05*	7.94 ± 0.03	8.8	−22.4
GmFAD2	10.5 ± 0.01*	6.81 ± 0.02*	3.68 ± 0.01*	76.46 ± 0.14*	4.96 ± 0.02*	5.73 ± 0.01*	11.5	−7.5
Triple	24.22 ± 0.01*	6.68 ± 0*	17.54 ± 0.01*	63.41 ± 0*	4.21 ± 0*	4.86 ± 0.02*	24.8 ± 6.8	2.6 ± 1.2

Percentage of total saturates (Sat). palmitic (16:0), stearic (18:0), oleic (18:1), linoleic (18:2), and linolenic acid (18:3), from wild type (WT), event 915‐25 carrying AtWRI1 (pPTN1174), event 683‐2 carrying mangosteen GmFatA1 (pPTN811), event 374‐1 carrying silencing element for GmFad2 (pPTN326), and triple stack extruded oil from seeds determined by GC analysis using TMSH derivatized oil samples (mean ± SD *n* = 3).

* Statistical differences across events. *P* < 0.05. Oxidative stability index displayed in hours (OSTI), and melting point (°C).

### Effect of interesterification on functionality of soybean oil high in saturated and oleic fatty acids

Soybean oil samples derived from the independent events 1008‐5, and 1026‐1, carrying the T‐DNA element of pPTN1289, and pPTN1301, respectively, were selected as a means to determine the impact of randomization of TAG species composition and crystallization behaviour of the oils. The samples were analysed for fatty acid composition using GC‐FID (Figure 3a), and for solid fat content (SFC) before and after randomization reaction using NMR (Figure 3b,c). Oils derived from the event 1008‐5 and 1026‐1 had a much higher solid content at 10 °C, melted in a much faster rate, and fully melted at a much lower temperature compared to the commercial margarine samples (Figure 3b,c). However, the melting profile of the two oils is different. The solid fat content at 10 °C is about 40% and 54% for 1026‐1 and 1008‐5, respectively. The complete melting point of 1026‐1 is about 24 **°**C, and that of 1008‐5 about 30 **°**C. Randomization reaction did not alter the SFC profile significantly.

## Discussion

A transcription factor approach was implemented to gain insight on the effects of the modulation of carbon partitioning between carbohydrate and lipids during embryogenesis in oil accumulation in soybean seeds. The outcome of introducing the *AtWRI1* into soybean, under the control of the seed specific β‐conglycinin, translated to an oil elevated in palmitate, with no discernable changes in total oil content under greenhouse or field environments. The monitoring of gene expression changes in selected gene calls, triggered by *AtWRI1*, associated with lipid biosynthesis, revealed an up‐regulation of Acyl‐CoA carboxylase (ACC), however, this outcome was insufficient to impact total oil levels in soybean seeds (Figure 2a). Given only ACC expression *per se* was monitored, and potential impacts on changes in fatty profile on feedback inhibition, posttranslationally on ACC, could explain why more carbon flux towards oil was not observed. The unexpected up‐regulation of palmitoyl‐ACP thioesterase B (*FatB*) observed in immature embryos expressing *AtWRI1*, led to increases in palmitate. The increase, up to nearly 20% of palmitate, was observed in plants grown under greenhouse, and under field conditions during 2015, 2016, and 2017 (Table 1, and Table S1). Expression analysis indicated that *AtWRI1* up‐regulates specifically the *FatB2a* allele in soybean seeds (Figure 2b,c), which is primarily expressed in flowers, and leaves, in a wild‐type soybean background (Cardinal *et al*., 2014).

AtWRI1 recognizes the AW‐box motif that resides upstream of multiple gene calls involved in lipid biosynthesis. The AW‐box motif has a consensus sequence [CnTnG](n)_7_[CG], where n can be any nucleotide (Maeo *et al*., 2009). *In silico* analysis was conducted to mine the *GmFATB* gene calls, including an approximate 1 kb upstream of the respective ATG, for presence of an AW‐box, employing the FIMO scan tool (www.meme-suite.org). The outcome of this exercise revealed the lack of an AW‐box in gene calls Glyma 05g012300 and Glyma 17g120400, but the *GmFATB* alleles, Glyma 04g151600 and Glyma 06g211300, contained three copies and one copy, respectively. However, in the latter cases the motifs found reside within the first exon of the gene calls, not 5’ to the ATG start of translation (Figure 2d). Hence, this *in silico* analysis does not easily explain the up‐regulation observed in the *GmFATB2a* allele when the *AtWRI1* transgenic allele is present in the genome. However, in mammalian systems motifs for transcription bindings sites have been reported to reside in first exon of gene calls, which are hypothesized to impact codon preference bias (Stergachis *et al*., 2013).

Altering the saturated fatty acid composition of seed oil may have pleotropic impact on other seed traits such as germination, which in turn will affect stand establishment under field environments. Monitoring of germination frequency, relative to WT seeds, revealed no significant changes in radical emergence at 25 °C, however, at 10 °C differences were observed (Figure S3). This information suggests that low soil temperature impacts on stand establishment are likely to be more profound on a soybean with such a fatty acid profile relative to WT. While stand counts were not a parameter that was assessed over the course of the field trials, no obvious reduction in stand establishment was noticed.

The end‐use functionality and nutritional properties of oils for food, feed, and industrial applications are influenced by the fatty acid composition in such oils (Clemente and Cahoon, 2009). A vegetable oil with high levels of saturated fatty acids, coupled with reduced polyunsaturated fatty acids, and elevated in oleic acid, possesses functionality attributes, due to improved oxidative stability and solid fat content (SFC), for both industrial and baking type applications (Anushree *et al*., 2017; Devi and Khatkar, 2016; Garcés *et al*., 2012; Graef *et al*., 2009; Knothe, 2005, 2008). As a means to exploit the high palmitate phenotype obtained by the expression of *AtWRI1* in soybean seeds as a potential contributor to increases in total SFC of the oil, transgene stacks were created with the goal of creating a soybean oil with high SFC and reduced polyunsaturated acids. This goal was first achieved through crossing strategy wherein a gene stacking approach, mirroring that previously communicated (Park *et al*., 2014), that brought together the AtWRI1 transgenic allele with a previously characterized soybean event harboring GmFatA1 allele, alone or combined with a silencing element targeting endogenous Fad2 gene (Buhr *et al*., 2002). The outcome of these stacks revealed that the combination of *AtWRI1* and *GmFata1*, increase SFC to approximately 28%, with a concomitant decrease in oleate, while the combination of *GmFatA1* and silenced *Fad2*, translated to elevated oleate and stearate, with a concurrent reduction in palmitate and polyunsaturates, primarily linolenic acid, while the triple stack brings back palmitate levels closer to wild‐type control (Table 2). The monitoring of oxidative stability and melting temperature of the various oils reflects the contribution of fatty acids levels on these two parameters, where reduction in polyunsaturated fatty acids is required for improvement in oxidative stability, while SFC and lowering of polyunsaturated fatty acids is necessitated for targeted outcome of improved oxidative stability and reduction in melting temperature (Table 4).

The data gleaned from the various transgene stacks created via crossing was subsequently used to inform genetic designs that would translate to phenotypic outcomes of high SFC, with elevated oleic acid in the seed oil. Here a single cassette was assembled, using a modification of a strategy previously communicated, that embeds a hair‐pin element within an intron that resides 5’ to the ORF of the cassette (Frizzi *et al*., 2008). The outcome of this learn/build step was the vectors pPTN1289 and pPTN1301 (Figure 1). Soybean events carrying pPTN1289 and pPTN1301 genetic elements produce oil with fatty acid profiles that tend to mirror the double stack (silenced *Fad2*/*GmFata1*) and triple stack, respectively (Tables 3, Table S4), with respect to overall changes in saturated and monounsaturated fatty acids levels in the oil. These oils possess a SFC at levels similar to many margarines type products currently on the market. However, the transition to a complete liquid, with or without interesterification, occurs at approximately 24 °C and 30 °C, for the pPTN1289 and pPTN1301 oils, respectively, while margarine products maintain SFC beyond 30 °C (Figure 3). It is unexpected that the randomization reaction did not alter the SFC profile significantly, particularly for 1008‐5. Typically for vegetable oils, the saturated fatty acids tend to occupy the sn‐1 and 3 positions of the glycerol backbone, and the more unsaturated fatty acids are more concentrated on sn‐2 position. After randomization, fatty acid composition at each sn‐position becomes identical, and more trisaturated molecular species should be produced, increasing the end melting point. However, the SFC result did not indicate such change. In the future, individual molecular species of the triacylglycerols should be analysed to study their composition in the native oils and the changes made by the reaction. Another direction is to fractionate the oil into a stearin and olein fractions and evaluate their functional properties and food applications separately. The fractional crystallization process can create valuable commercial products, such as plastic fats for baking and stable oils for frying, just as what is being done for the palm oil. Hence, the novel soybean oils communicated herein likely will require interesterification with other hardstocks to be more desirable oils in baking applications and other end use scenarios that require high SFC coupled with reduced polyunsaturated fatty acids.

When the AtWRI1 cassette is combined with the mangosteen FatA1, and silenced Fad2‐1 transgenic alleles, stearic acid level is approximately 13%, oleic acid is close to 60%, and palmitic acid is restored to approximately 9%, with the exception of data obtained from 2015 harvest (Table 4). In addition, the resultant oil displayed low percentage of polyunsaturated fatty acids (Tables 2 and 3, Tables S2–S4). Similar efforts that combined the *fad2‐1a*,* fad2‐1b*,* fad3a,* and *fad3c* alleles produced a soybean oil high in oleate and low in linolenate close in fatty acid composition to that of olive oil (Pham *et al*., 2012). Also, crossing high palmitic with high steric soybean lines produced an oil with a total of 38% saturates (Rahman *et al*., 2003), whereas overexpression of a mangosteen FatA1, with the down‐regulation of FatB and FAD2*‐1* led to soybeans seed oil with reduced palmitate and increased stearate, and oleate (Park *et al*., 2014) similar to the sunflower oil profile obtained through conventional breeding (Perez‐Vich *et al*., 2002). Our strategy for the assembly of genetic constructs linked to a single T‐DNA using the tools of biotechnology allowed for the reduction in copies of selectable markers, and helped minimize the duplication of genetic elements introduced into the soybean genome without compromising the phenotypes observed by crossing.

The synthesis of a high SFC, low PUFA soybean oil communicated herein is yet another example of how the tools of biotechnology can serve as a conduit to introduce novel bits of genetic variation to complement plant breeding programs. The challenges that must be overcome to see this and other agronomic output traits, that require some level of identity preservation, translating to the marketplace, include a creation of a global regulatory framework that focuses on the trait and not the process by which the trait is created (CAST, 2018), and second, building of infrastructure and supply chains to enable cost effective pipeline from the farmgate to the consumer.

## Experimental procedures

### Vector construction and plant transformation

The Arabidopsis *AtWRI1* (GenBank accession NP_191000) was synthesized and codon optimized for soybean (GenScript, Piscataway, NJ), and fused to the tobacco etch virus translational enhancer element (Carrington and Freed, 1990). The plasmid referred to as pPTN1174 has the *AtWRI1* under the control of the soybean seed‐specific β‐conglycinin promoter (Allen *et al*., 1989), and terminated with the 35S cauliflower mosaic virus polyadenylation signal. The binary vectors pPTN326 and pPTN811, harboring a soybean Δ‐12 fatty acid desaturase *FAD2‐1* (*GmFAD2‐1*) silencing element and a mangosteen stearoyl‐ACP thioesterase (*GmFatA1*), respectively, were previously communicated (Buhr *et al*., 2002; Park *et al*., 2014). Two constructs were then assembled to carry a dual *GmFAD2‐1* silencing element/*GmFatA1* expression cassette, alone or combined with the *AtWRI1* cassette described above. The expression of the dual element was driven by the seed‐specific phaseolin promoter, fused to the tobacco etch virus translational enhancer element, and terminated by the *Agrobacterium* nopaline synthase polyadenylation signal. Each resultant cassette was subcloned into a binary vector which harbors a *bar* gene (Thompson *et al*., 1987) under control of the *Agrobacterium* Pnos promoter for selection. The resultant vectors, designated pPTN1289 and pPTN1301, respectively, were mobilized independently into *A. tumefaciens* strain EHA101(Hood *et al*., 1986) via triparental mating. Soybean transformations were conducted using the resultant transconjugants following the procedures previously described (Xing *et al*., 2000; Zhang *et al*., 1999).

### Generation of gene stacks

Gene stacks were generated to increase total saturates and oleic acid in transgenic soybean. Crosses were made by crossing high palmitate pPTN1174 events designated 915‐25, or 917‐17, with transgenic events that displays increased stearic acid referred to as 682‐2, or 687‐2, which carry a mangosteen *GmFatA1* (Park *et al*., 2014); and with an event displaying high oleic acid referred to as 374‐1, which carries a silencing element for *GmFad2* (Buhr *et al*., 2002). Double stacks were generated by crossing 915‐25 × 683‐2, 917‐17 × 683‐2, 917‐17 × 687‐2, and 374‐1 × 687‐2. Triple gene stack was generated by crossing the double stack ‘915‐25 × 683‐2’ with 374‐1. Selected double and triple stacks were advanced to homozygousity.

### Selection of transgenic plants

Identification of transformed plants was determined by monitoring the expression of the *bar* gene via leaf painting technique (Zhang *et al*., 1999). The presence of *AtWRI1*,* GmFatA1*, or *GmFad2* in transgenic plants was monitored via PCR. Genomic DNA was isolated following a modified CTAB method (Springer, 2010). Synthetic *AtWRI1* was amplified with the primer set 5′‐ATGGGCATGAAAAAGAGACTTACTACATCC‐3′and 5′‐TTATTCTGAGCCAACGAAGAGACCCT G‐3′. Amplification of *GmFatA1* was carried out with the primer set 5′‐ATGGCACTTAAACTCTCCTCATCCAGAAG‐3′ and 5′‐CTATCT TGTTGGTTTCTTCCTCCACTCAG‐3′. Amplification of *GmFad2* silencing element was done with the primer set 5′‐CAACCCACACACAAACACATTGCCTTTTTC‐3′,and 5′‐CCTAGAGGGTTGTTTAAGTACTTGGAAAACC‐3′. The PCR reactions were conducted with Go Taq ^®^ Green Master Mix, following the manufacturer's instructions (Promega Corporation, Madison, WI).

### Molecular characterization of transgenic events

Southern blot hybridization was used to determine transgenic allele integration. Genomic DNA was isolated from young leaves according to the protocol described by Dellaporta *et al*., (1983). Ten micrograms of genomic DNA were digested with the restriction enzymes *Bgl II,* or *Hind III,* from events harboring pPTN1174, which has a single restriction site within the T‐DNA. Genomic DNA from events harboring pPTN1289, and pPTN1301 were digested with *Hind III*, and *BamH I*, respectively. The DNA samples were separated via electrophoresis on 1% agarose gels. The samples were blotted and UV crossed linked to a nylon membrane (Bio‐Rad cat #162‐0196, Hercules, CA). Membranes were hybridized with dCT^32^P labelled region of either *AtWRI1* or *Bar* ORF for pPTN1174 events, and a region of the *GmFatA1* ORF for pPTN1289, and pPTN1301 events using random prime labelling (Prime‐It II Cat # 300385; Stratagene, La Jolla, CA) as previously described (Eckert *et al*., 2006).

### Gene expression analyses

Gene expression of *AtWRI1* and target genes was determined via northern blot hybridization in transgenic events harboring pPTN1174. RNA was isolated from immature embryos harboring pPTN1174 using TRIzol ^®^ reagent (Invitrogen, Carlsbad, CA, cat. # 10296‐028) following the manufacturer's protocol. RNA quality and concentration were determined with NanoDrop^®^ ND 1000 Spectrophotometer (Thermo Fisher, Waltham, MA). Fifteen micrograms of RNA were separated by electrophoresis on 1% agarose gels. The samples were blotted, and UV cross‐linked to a nylon membrane (Bio‐Rad cat #162‐0196). Membranes were hybridized with dCT^32^P labelled region of either AtWRI1, or endogenous Pyruvate Kinase, Acetyl CoA Carboxylase, Acyl Carrier Protein, and Keto‐ACP synthase genes were used as a probe using random prime labelling (Prime‐It II Cat # 300385; Stratagene) as previously described (Eckert *et al*., 2006).

### RT‐PCR, and qRT‐PCR analysis

Gene expression of four *GmFatB* alleles were quantified in selected events carrying the *AtWRI1* cassette. RNA was isolated from immature embryos using RNeasy Plant mini kit (Qiagen, Venlo, Netherlands) following the manufacturer's instructions. RNA quality and concentration were determined with NanoDrop^®^ ND 1000 Spectrophotometer (Thermo Fisher). cDNA was synthesized from 3 μg RNA using SuperScript III First Strand kit following manufacturer's protocol (Cat # 18080‐051; ThermoFisher Scientific). Primers sets were designed against unique regions of 200 bp of the 3′UTR of the different *GmFatB* genes. *GmFATB2a* (Glyma.04g151600) was amplified with: 5′‐GAGAGCTTTGCTTGTTTTTATCAAATCTACGTATC‐3′,and 5′‐CCATAAGTGAAACAGAAAATGGAGCAGGTAG‐3′. *GmFATB2b* (Glyma.06g211300) was amplified with 5′‐CATGTTCCCACTTGCAGATTGGAGAGC‐3′, and 5′‐AAGAACTATAATATACACGCTTAGCCATTTTTATGAAACAATT‐3′. *GmFATB1a* (Glyma.05g012300) was amplified with 5′‐CATTCTCATTGTAATTAGCTACTGCTGTATTCTCTC‐3′, and 5′‐GAATAAGAATTATGCTGTGTTCACAAGGAAAATTGC‐3′. *GmFATB1b* (Glyma.17g120400) was amplified with 5′‐CCTCATTCTCTCTTTCTGCTGCTCCATATTTC‐3′,and 5′‐CACGGTTGTATTGGATGGACAAGTCATTC‐3′. Actin (*GmSAc7* Glyma.08G146500) was used as a reference and amplified 5′‐GAGTCTGGCCCATCCATTGTCCAC‐3′, and 5′‐GGCCCCCACACAAAATATGATGCATCAAG‐3′. The RT‐PCR reactions were conducted with Go Taq ^®^ Green Master Mix, following the manufacturer's instructions (Promega Corporation). Quantitative real time PCR (qRT‐PCR) was performed in a BioRad iCycler using iTaq^™^ Universal SYBR^®^ Green Supermix (Cat # 172‐5121). Soybean actin gene was used as internal reference to normalize Ct‐values obtained for each GmFatB gene. Data were analysed using the methods described by Dussault and Pouliot (2006).

### Fatty acid analysis

Fatty acid profiles of transgenic events were determined on soybean seeds or cotyledon chips via gas chromatography following the procedures previously described (Buhr *et al*., 2002; Cahoon *et al*., 2001). Derived methyl esters were analysed on a 6890N gas chromatography‐flame ionization detector (Agilent Technologies, Santa Clara, CA) fitted with a 30‐m × 250 μm HP‐INOWAX column (Cat # 19091N‐133; Agilent Technologies). Fatty acids were reported as percentages of total fatty acids were analysed using either *t*‐Test or ANOVA.

### Field evaluation of transgenic events

Field trials were conducted on homozygous transgenic events and lineages. Events harboring *AtWRI1* and stacks were evaluated during 2015, 2016, and 2017. Transgenic events harboring pPTN1289 and pPTN1301 were evaluated during 2016, and 2017. All evaluations were conducted in completely randomized plots. Each plot consisted of four 10 ft rows. Data were collected from the plants in the inner two rows. Either two or three plots per event/lineage were planted each year depending on the seed availability. Fatty acid profiles were determined as described above. Oxidative stability, cloud and melting points were monitored on soybean oil extruded from homozygous transgenic events and lineages growing under field conditions following procedures previously described (Park *et al*., 2014). ANOVA was conducted for statistical analysis.

### Statistical analysis of data

All fatty acid data collected from field plots were analysed as complete randomized designs using analysis of variance (ANOVA) procedures of SAS 9.3 software (SAS Institute Inc. Cary, NC). Given seed availability each year, either two or three plots per event/lineage were planted. Each plot consisted of four 3.1 meter rows. For events derived from pPTN1174, pPTN1289, and pPTN1301 data were collected from seven bulked seed per plant, obtained from two plants grown within the inner two rows. Data from the stacks made by crossing, grown under field conditions, were collected from 40 bulked seeds/stack per plot. Statistical analysis of the fatty acid data collected from events/lineages grown under greenhouse conditions were analysed by *t*‐tests using GraphPad Prism 6 software (La Jolla, CA).

### Physical and biochemical properties of derived oils

Randomization of the test oils (15 g) was performed at 60 **°**C for 2 h with the addition of dried sodium methoxide (1% w/w). Citric acid monohydrate was added to stop the reaction, and the samples were rinsed with distilled water several times to remove impurities. For solid fat content measurements, the oils were heated and mixed and transferred to NMR tubes. The tubes were stored overnight at 4 **°**C before oils were measured for SFC at 10, 20, 30, 40, and 50 **°**C. The tubes were incubated at each temperature for 30 min before measurements were taken. MS and MT are margarine sticks and tub of commercial sources used for comparisons.

## Author contributions

PV, TEC and SBN, and EBC conducted research and data analysis. HP, HN, and LH conducted research. HN generated transgenic events and contributed to molecular analysis and generation of gene stacks. HLK, TW, and ONC contributed to characterization of oils. PV, SB, and TEC wrote the manuscript. EBC and TEC supervised project activities.

## Conflict of interest

The authors declare that the research was conducted in the absence of commercial or financial ties that could lead to any conflict of interest.

## Supporting information


**Figure S1** Southern blot analysis of pPTN1174 soybean events.
**Figure S2** Southern blot analysis of pPTN1289 and pPTN1301 soybean events.
**Figure S3** Effect of temperature on seed germination in transgenic events1022‐12 (pPTN1289), 1026‐1 (pPTN1301), and WT.
**Table S1** Fatty acid profile of soybean seeds carrying AtWRI1.
**Table S2** Fatty acid profile of soybean events and stacks.
**Table S3** Fatty acid profile of soybean events and stacks evaluated under field conditions.
**Table S4** Fatty acid profile of T2 seeds from pPTN1289 and pPTN1301 events grown under greenhouse conditions.
