# Supplementary material for: Expression of the Arabidopsis WRINKLED 1 transcription factor leads to higher accumulation of palmitate in soybean seed
Source: Plant Biotechnol J. 2019 Jan 18;17(7):1369–79. doi: 10.1111/pbi.13061 (PMC6577354; doi:10.1111/pbi.13061)
Supplement: Supplementary file 1 — Figure S1 Southern blot analysis of pPTN1174 soybean events. Figure S2 Southern blot analysis of pPTN1289 and pPTN1301 soybean events. Figure S3 Effect of temperature on seed germination in transgenic events1022‐12 (pPTN1289), 1026‐1 (pPTN1301), and WT. Table S1 Fatty acid profile of soybean seeds carrying AtWRI1. Table S2 Fatty acid profile of soybean events and stacks. Table S3 Fatty acid profile of soybean events and stacks evaluated under field conditions. Table S4 Fatty acid profile of T2 seeds from pPTN1289 and pPTN1301 events grown under greenhouse conditions. [file PBI-17-1369-s001.docx]

Pamela A. Vogel^1,2*^, Shen Bayon de Noyer^1,2*^, Hyunwoo Park^1,2#^, Hanh Nguyen^3^, Lili Hou^1,2^, Taity Changa^1,2^, Hoang Le Khang^1,2^, Ozan N. Ciftci^4^, Tong Wang^5^, Edgar B. Cahoon^1,6^, and Tom Elmo Clemente^1,2^

^1^ Center for Plant Science Innovation, University of Nebraska-Lincoln, Lincoln, NE, 68588.

^2^ Department of Agronomy & Horticulture, University of Nebraska-Lincoln, Lincoln, NE, 68583

^3^ Center for Biotechnology, University of Nebraska-Lincoln, Lincoln, NE, 68588.

^4^ Department of Food Science & Technology, University of Nebraska-Lincoln, Lincoln, NE 68588

^5^ Department of Food Science and Human Nutrition, Iowa State University, Ames, IA, 50011.

^6^ Department of Biochemistry, University of Nebraska-Lincoln, Lincoln, NE 68588

^#^ Current address: LG Chem, Seoul, Republic of Korea

*These authors contributed equally to this work

Corresponding author: Tom Elmo Clemente, Department of Agronomy and Horticulture, Center for Plant Science Innovation. University of Nebraska-Lincoln, Lincoln, NE, 68588, USA. Phone: +1 (402) 472-1428. Fax: +1 (402) 472-3139.

Email: [tclemente1@unl.edu](mailto:tclemente1@unl.edu)

**Supplemental materials**

**Figure S1.** Southern blot analysis of pPTN1174 soybean events. Genomic DNA was hybridized to a region of the (a) AtWRI1, or (b) bar gene. Lane 1: 915-25. Lane 2: 917-17, Lane 3: 917-26, Lane 4: WT and, Lane 5: pPTN1174 for positive control.

**Figure S2.** Southern blot analysis of pPTN1289 and pPTN1301 soybean events. Genomic DNA was hybridized to a region of the GmFatA1 gene.

**Figure S3.** Effect of temperature on seed germination in transgenic events1022-12 (pPTN1289), 1026-1 (pPTN1301), and WT. Germination rate of transgenic events was determined by number of radicles emerging from testa from 25 (T_2_) seeds per day, during 17 days after imbibition at (a) 10°C, and (b) 25°C. (n=4).

| **Table S1**. Fatty acid profile of soybean seeds carrying AtWRI1 | | | | |  |
| --- | --- | --- | --- | --- | --- |
| **Event** | **16:0** | **18:0** | **18:1** | **18:2** | **18:3** |
| WT | 11.1 ± 0.2 ^c^ | 3 ± 0 ^bc^ | 17.5 ± 1 ^a^ | 53.1 ± 0.7 ^b^ | 12.8 ± 0.8 ^a^ |
| 915-25 | 18.8 ± 0.4 ^a^ | 2.7 ± 0.1 ^c^ | 9 ± 0.2 ^c^ | 55 ± 0.8 ^a^ | 11.5 ± 0.4 ^a^ |
| 917-17 | 18 ± 0.8 ^a^ | 3.1 ± 0.1 ^b^ | 9.4 ± 0.4 ^c^ | 53.6 ± 0.3 ^ab^ | 13.2 ± 0.6 ^a^ |
| 917-26 | 15.1 ± 1.3 ^b^ | 3.3 ± 0.1 ^a^ | 11.7 ± 0.6 ^b^ | 54.5 ± 0.3 ^ab^ | 13.2 ± 0.7 ^a^ |
| Percentage of palmitic (16:0), stearic (18:0), oleic (18:1), linoleic (18:2), and linolenic acid (18:3), in soybean events harboring WRI1 (pPTN1174). Plants were grown under greenhouse conditions. Data expressed as mean ± standard error (n=8). Different letters indicate statistical differences (p<0.05) within events. | | | | | |

| **Table S2.** Fatty acid profile of soybean events and stacks. | | | | | | |
| --- | --- | --- | --- | --- | --- | --- |
| **Gene** | **Event** | **16:0** | **18:0** | **18:1** | **18:2** | **18:3** |
| WT | WT | 12.3 ± 0.1 | 3.5 ± 0 | 12.2 ± 0.3 | 53.4 ± 0.7 | 16 ± 1.1 |
| AtWRI | 915-25 | 19.3 ± 0.2* | 2.9 ± 0.2 | 8.4 ± 0.1 | 54 ± 0.6 | 12.8 ± 0.5* |
| AtWRI | 917-17 | 18.5 ± 1* | 3.1 ± 0.1* | 8.2 ± 0.3 | 52.3 ± 0.8 | 15.6 ± 0.6 |
| GmFATA1 | 683-2 | 7.3 ± 0.1* | 21.7 ± 0.3* | 9 ± 0.5 | 40.6 ± 1.1* | 14.6 ± 0.4 |
| GmFATA1 | 687-2 | 11.2 ± 0.2 | 6.3 ± 0.5* | 15 ± 1.2 | 51.2 ± 0.5 | 13.5 ± 0.8* |
| RNAi FAD2-1 | 374-1 | 8.1 ± 0.2* | 3.2 ± 0.1 | 74.1 ± 0.9* | 3.8 ± 0.6* | 7.1 ± 0.3* |
| RNAi FAD2-1 x GmFATA1 | 374-1 x 687-2 | 6.7 ± 0.3* | 5.8 ± 0.6* | 75.4 ± 0.5* | 2 ± 0.1* | 6.3 ± 0.2* |
| AtWRI x GmFATA1 | 917-17 x 687-2 2 | 18.3 ± 1* | 8.2 ± 1.4* | 10.1 ± 0.3 | 49.5 ± 0.6 | 11 ± 0.5* |
| AtWRI x GmFATA1 | 917-17 x 687-2 3 | 16 ± 1.1* | 12.4 ± 1.9* | 9.2 ± 0.3 | 49.6 ± 1 | 10 ± 0.4* |
| GmFATA1 x WRI | 683-2 x 915-25 | 11 ± 0.5 | 21.4 ± 1* | 9.3 ± 0.4 | 43.7 ± 1.1* | 11.2 ± 0.6* |
| RNAi FAD2-1 x GmFATA1 x AtWRI | 374-1 x 683-2 x 915-251 | 10.3 ± 0.7 | 29.7 ± 0.8* | 27.9 ± 6.8* | 20.5 ± 6.5* | 7.8 ± 0.5 * |
| Percentage of palmitic (16:0), stearic (18:0), oleic (18:1), linoleic (18:2), and linolenic acid (18:3) in soybean seeds harboring AtWRI1 (pPTN1174), mangosteen GmFatA1 (pPTN811), RNAi GmFad2 (pPTN326), double and triple stacks derived by crossing. TMSH derivatization was performed separately on chips of 6 seeds from homozygous plants grown under greenhouse conditions. Data indicates mean ± stdev (n=6). *Statistical differences across events. p<0.05 | | | | | | |

| **Table S3.** Fatty acid profile of soybean events and stacks evaluated under field conditions | | | | | | |
| --- | --- | --- | --- | --- | --- | --- |
| **Gene** | **Event** | **16:0** | **18:0** | **18:1** | **18:2** | **18:3** |
| WT | WT | 11.0±0.5 | 3.0±0.2 | 18.1±2.8 | 53.1±1.9 | 12.6±1.6 |
| AtWRI | 915-25 | 19.3±0.6* | 2.3±0.2 | 13.0±1.6* | 54.6±1.4 | 8.7±0.9* |
| GmFATA1 | 683-2 | 7.3±0.4* | 18.3±1.3* | 14.2±1.7* | 44.6±1.4* | 11.6±1.2 |
| RNAi FAD2-1 | 374-1 | 7.2±0.3* | 2.8±0.2 | 77.2±1.8* | 3.2±0.7* | 6.9±0.9* |
| GmFATA1xAtWRI | 683-2 x 915-25 | 11.5±0.5 | 17.6±0.4* | 10.9±0.8* | 48.4±0.9* | 8.5±1.1* |
| RNAi FAD2-1xGmFATA1 | 374-1 x 687-2 | 5.4±0.4* | 8.6±1.3* | 72.6±2.4* | 3.4±0.9* | 6.9±1.0* |
| RNAi FAD2-1xGmFATA1xAtWRI | 374-1 x 683-2 x 915-25 | 8.5±1.3* | 19.1±2.1* | 57.3±4.8* | 4.6±1.9* | 6.7±1.0* |
| Percentage of palmitic (16:0), stearic (18:0), oleic (18:1), linoleic (18:2), and linolenic acid (18:3) in soybean seeds harboring AtWRI1 (pPTN1174), mangosteen GmFatA1 (pPTN811), RNAi GmFad2 (pPTN326), double and triple stacks derived by crossing. TMSH derivatization was performed separately on seed chips from homozygous plants grown under field conditions. Data indicates mean ± stdev (n=8). *Statistical differences across events. p<0.05 | | | | | | |

| **Table S4.** Fatty acid profile of T2 seeds from pPTN1289 and pPTN1301 events grown under greenhouse conditions | | | | | | | |
| --- | --- | --- | --- | --- | --- | --- | --- |
| **Plasmid** | **Event** | **Total Saturates** | **16:0** | **18:0** | **18:1** | **18:2** | **18:3** |
| WT | WT | 15.6±0.8 | 12.2±0.7 | 3.4±0.3 | 12.8±1.5 | 55.5±1.4 | 13.7±1.2 |
| pPTN1289 | 1008-5 | 23.2±2.4* | 3.8±0.2* | 19.4±2.3* | 65.4±2.0* | 1.2±0.1* | 3.7±0.5* |
| pPTN1289 | 1008-6 | 29.0±3.7* | 4.1±0.5* | 24.9±3.4* | 60.6±2.8* | 1.3±0.3* | 3.3±0.5* |
| pPTN1289 | 1022-3 | 25.2±1.9* | 4±0.4* | 21.2±1.9* | 65.4±2.8* | 1.1±0.3* | 3.7±0.7* |
| pPTN1289 | 1022-4 | 19.9±1.3* | 4.4±0.6* | 15.5±1.5* | 69.6±1.8* | 1.6±0.3* | 4.3±0.5* |
| pPTN1289 | 1022-15 | 28.4±1.8* | 3.9±0.2* | 24.6±1.7* | 62.0±1.7* | 1.1±0.2* | 4.0±0.5* |
| pPTN1301 | 1026-1 | 29.2±1.5* | 6.6±0.4* | 22.6±1.7* | 60.1±1.4* | 1.5±0.2* | 4.2±0.6* |
| pPTN1301 | 1028-3 | 23.7±2.9* | 11.7±0.4 | 12.1±3.2* | 58.4±1* | 7.7±3.2* | 7.6±0.7* |
| pPTN1301 | 1018-4 | 23.7±3* | 9.3±2.3* | 14.4±4.3* | 65.8±3.3* | 2.1±0.9* | 5.1±1.2* |
| pPTN1301 | 1007-12 | 22.6±1* | 10.3±0.8* | 12.4±1.2* | 59.5±3.1* | 6.5±1.9* | 8.6±1.3* |
| pPTN1301 | 1028-1 | 22.2±1.1* | 12.1±0.4 | 10.1±0.9* | 55±2* | 12.1±3.4* | 8.3±0.3* |
| Percentage of total saturates, palmitic (16:0), stearic (18:0), oleic (18:1), linoleic (18:2), and linolenic acid (18:3) in soybean seeds harboring pPTN1289, and pPTN1301. TMSH derivatization was performed separately on seed chips from plants grown under greenhouse conditions. Data indicates mean ± stdev (n=8). *Statistical differences p<0.05 | | | | | | | |
